# Maintaining RNA Integrity for Transcriptomic Profiling of Ex Vivo Cultured Limbal Epithelial Stem Cells after Fluorescence-Activated Cell Sorting (FACS)

**DOI:** 10.1186/s12575-017-0065-2

**Published:** 2017-12-12

**Authors:** Lei Liu, Frederik Mølgaard Nielsen, Simone Elkjær Riis, Jeppe Emmersen, Trine Fink, Jesper Østergaard Hjortdal, Chris Bath, Vladimir Zachar

**Affiliations:** 1 Laboratory for Stem Cell Research, Aalborg University, Aalborg, Denmark; 2 Department of Pediatric Surgery, First Hospital of Jilin University, Changchun, China; 3 Department of Ophthalmology, Aarhus University Hospital, Aarhus, Denmark; 4 Department of Ophthalmology, Aalborg University Hospital, Aalborg, Denmark

**Keywords:** Limbal stem cells, RNA integrity, Fluorescence-activated cell sorting, RNA sequencing, Intracellular staining, Ethanol

## Abstract

**Background:**

Transcriptomic profiling of ex vivo cultured human limbal epithelial stem cells (hLESCs) will foster better understanding of corneal physiology and novel treatment paradigms to limbal stem cell deficiency (LSCD). However, currently such profiling studies are hampered due to difficulties with producing sufficient amounts of intact mRNA for deep RNA sequencing (RNA-seq) from subpopulations sorted on the basis of co-expression of membrane and intracellular antigens by fluorescence-activated cell sorting (FACS).

**Methods:**

To address this problem, we systematically analyzed the critical steps, and found that ethanol fixation together with optimized downstream procedures provided a pipeline that yielded high quality total RNA in amounts to readily support the RNA-seq procedure, while still preserving good discrimination between the individual hLESC immunophenotypes.

**Results:**

The average RNA integrity number (RIN) was 7.7 ± 0.4, and the average yield was 4.6 ± 1.7 pg of RNA per cell. The sequencing analysis of the isolated RNA produced high quality data with more than 70% of read pairs mapping uniformly to the reference genome and 80% of bases with a Phred score of at least 30.

**Conclusion:**

In this study, we developed a reliable FACS-based procedure using ethanol as a fixative that would support accurate isolation of limbal epithelial progenitor subpopulations along with RNA yield and quality sufficient to enable deep transcriptomic profiling.

**Electronic supplementary material:**

The online version of this article (10.1186/s12575-017-0065-2) contains supplementary material, which is available to authorized users.

## Background

Corneal epithelial disease due to limbal stem cell deficiency (LSCD) is a major cause of blindness worldwide [1]. The use of ex vivo expanded human limbal epithelial stem cells (hLESCs) for transplantation [2] has been a remarkable success, however with a failure rate of 30% there is still room for improvement [3]. Previously, advanced techniques, including laser capture microdissection and deep RNA sequencing (RNA-seq), provided an unprecedented level of detail of molecular networks involved in the regulation of different limbal niche compartments in vivo [4]. Similar level of information regarding the cultured hLESCs is needed to understand how the ex vivo phenotype departs from the in vivo counterpart, and, importantly, to devise procedures whereby hLESCs can be expanded while preserving the properties they had in situ. To this end, a detailed transcriptomic analysis of specific hLESC lineages is warranted.

To achieve this goal, defined hLESC subpopulations need to be identified and isolated, and ultimately yield a high quality RNA. In the face of difficulties to identify an archetypal stem cell profile, the stemness in the cultured hLESCs is currently believed to be associated with the surface ATP-binding cassette sub-family B member 5 (ABCB5) [5] and/or intranuclear transformation-related protein 63 (p63) [6] antigens, in the absence of the cytoplasmic differentiation marker cytokeratin 3 (CK3) [7]. Consequently, any sorting attempt is contingent on a complex immunostaining procedure for both extra- and intracellularly located molecules that must yield accurate and reliable discrimination of different epitopes, and simultaneously preserve the RNA.

The fixation and permeabilization steps have in particular been shown to have an adverse effect on the RNA integrity [8–10], but the sorting procedure also presents a formidable obstacle due to challenges with securing an RNase-free environment and minimizing the physical stress imposed on the cells [11–13].

There is only scanty literature dealing with transcriptomic profiling of cells sorted by FACS on the basis of simultaneous surface and intracellular immunostaining. Incidentally, most of these reports are based on the use of formaldehyde as a fixative [14–17] but also methanol [18]. Our own results unfortunately could not lend credence to the usefulness of these fixatives in similar scenarios due to a high level of RNA degradation (unpublished data). Along these lines, evidence has previously been produced to show that formaldehyde results in a decrease of RNA integrity when used for transcriptional analysis of histological specimens [8–10]. The formation of irreversible cross-links between nucleic acids and proteins is believed to play a major role [8]. To avoid cross-linking, ethanol has been assayed, and turned out to compare satisfactory in terms of RNA yield and integrity [19–21]. Ethanol thus appears as a good candidate in a complex setup, where high quality RNA needs to be obtained in the face of multistep procedure involving fixation, permeabilization, labeled antibodies, and FACS sorting. Since there is no evidence that such trial would be carried out previously, we attempted to invoke ethanol in conjunction with all other specific steps to outline a novel pipeline to support RNA-seq and applied it to cultured hLESCs.

## Methods

### Cell Culture

The hLESCs isolation and culture protocol was optimized based on our previous report [22]. Human corneal scleral ring remnants (donors aged 22–86 years, 64% were men, absence of corneal disease) after keratoplasty procedures were obtained from the Department of Ophthalmology, Aarhus University Hospital (Arhus, Denmark), and handled according to Danish legislature. In brief, the rings from 10 to 12 randomly chosen donors were first rinsed with sterile phosphate buffered saline (sPBS, Gibco™, Life Technologies, Naerum, Denmark) supplemented with 100 units/ml penicillin and 100 μg/ml streptomycin (Life Technologies). After scraping off the endothelial side, the rings were incubated with 2.4 units/ml dispase II (Life Technologies) in sPBS at 37 °C for 1 h. Cells were then removed from the epithelial side, pooled briefly and pelleted at 300 g for 5 min, incubated with TrypLE Express (Life Technologies) at 37 °C for 8 min, and finally purified through a 70 μm cell-strainer (BD Biosciences, San Jose, CA). The resulting cell suspension was seeded directly into a T25 culture flask (Corning CellBIND; Sigma–Aldrich, Copenhagen, Denmark) at a density of 1000 to 1500 cells/cm^2^ in complete Keratinocyte-SFM (Life Technologies). The medium was changed every 2–3 days. At 80–90% confluency, the cells were trypsinized using TrypLE Express at 37 °C for 10 min, filtered through strainer, washed and resuspended in sPBS. Single cell suspensions of 0.5 to 1.5 × 10^6^ cells were aliquoted into 2.0 ml RNase-free microfuge tubes (Ambion, Naerum, Denmark) for subsequent immunofluorescence staining. The isolation and expansion process were repeated at 3 distinct time points.

### Fixation and Permeabilization

**Fig. 1:**
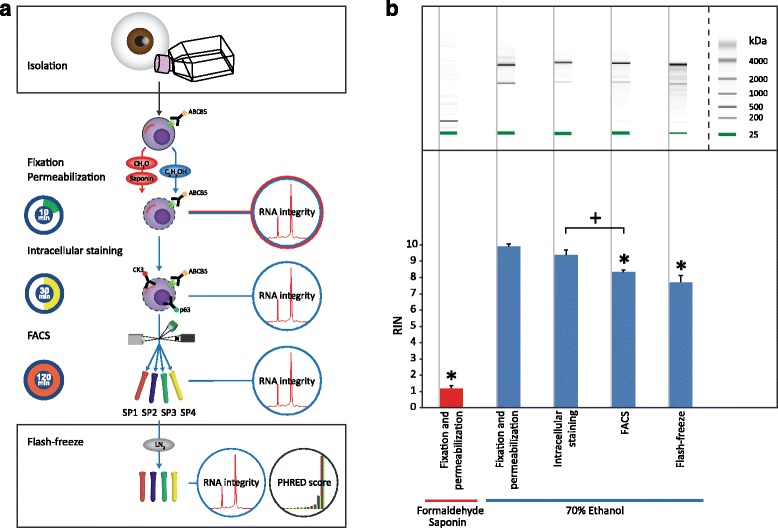
Effect of immunostaining and FACS on the integrity of RNA. **a** Flow chart of the staining and sorting procedures indicating the critical steps during the FACS-mediated isolation of the subpopulations SP1–4 and the steps at which RNA integrity was analyzed. Flash-freeze indicates samples flash frozen in liquid nitrogen and stored at −80 °C after the sorting. **b** Qualitative integrity of total RNA from the discrete steps during the immunostaining and sorting procedure. The quantitative data are presented as mean ± SD (*n* = 2–4). * denotes a significant (*p* < 0.05) lower RIN when compared to RIN directly after fixation and permeabilization with 70% ethanol, † denotes a significant lower RIN by pairwise comparison. ABCB5 = ATP-binding cassette sub-family B member 5; CH2O = formaldehyde; C2H5OH = ethanol; CK3 = cytokeratin 3; p63 = transformation-related protein 63; LN_2_ = liquid nitrogen; RIN = RNA integrity number; FACS = fluorescence-activated cell sorting

For optimization, two fixation and permeabilization methods were compared. The first method included fixation with 4% formaldehyde (Applichem, Esbjerg, Denmark) at room temperature for 15 min and subsequent permeabilization with 0.1% saponin (Sigma–Aldrich) in PBS supplemented with 1:100 Rnasin Plus Rnase inhibitor (Promega, Roskilde, Denmark) at 4 °C for 30 min. The second method was a one-step fixation and permeabilization using 70% ethanol in PBS (Ethanol absolute Electran® Molecular biology grade, VWR, Søborg, Denmark) at 4 °C for 10 min (Fig. 1a).

### Immunofluorescence Staining

The cells were first stained in suspension with direct conjugated antibody against the surface marker ABCB5 (for more information on antibodies, see Additional file 1), followed by washing with sPBS includes 50% Accumax (Sigma-Aldrich), and 25 mM HEPES (Life Technologies) at 300 g at 4 °C for 3 min and fixation and permeabilization with 70% ethanol at 4 °C for 10 min. After further washing, directly conjugated antibodies against intracellular p63 and CK3 were applied, and the staining procedure was concluded by two additional washing steps. As negative controls, non-specific isotype matching antibodies were used for p63 and CK3, and fluorescence minus one (FMO) for ABCB5. The antibodies were used in optimal dilution in PBS with 0.1% BSA (Sigma-Aldrich) and 50% Accumax, and 25 mM HEPES. After fixation and permeabilization, the staining and washing buffers were supplemented with Rnasin Plus Rnase Inhibitor diluted 1:10 and 1:100, respectively. All staining steps were performed in the dark at 4 °C for 30 min, and the volumes were kept at 100 μl for 10^6^ cells. In order to discriminate dead cells, the LIVE/DEAD Fixable Dead Cell Stain Kit (Life Technologies) was used according to the manufacturer’s instructions. Following staining, the cells were resuspended in a sort/collect buffer consisting of the washing buffer with 10-fold diluted RNasin Plus Rnase Inhibitor in a concentration to yield from 0.5 to 2 × 10^6^ cells per ml. The cell suspensions were then filtered through the Pre-Separation Filters (Miltenyi Biotec, Bergisch Gladbach, Germany) into 5 ml round bottom polystyrene tubes (BD Falcon, Albertslund, Denmark). All samples were left on ice in the dark until FACS.

### FACS

The flow cytometric analysis and sorting were performed using a MoFlo Astrios cell sorter (Beckman Coulter, Brea, CA). In order to avoid RNase contamination, the instrument was thoroughly decontaminated with a cleaning agent (COULTER CLENZ; Beckman Coulter), RNaze ZAP (Sigma-Aldrich), 70% ethanol, and milli-Q water. After decontamination, RNaseAlert Lab Test kit (Ambion) was used to ensure the complete absence of RNase.

**Fig. 2:**
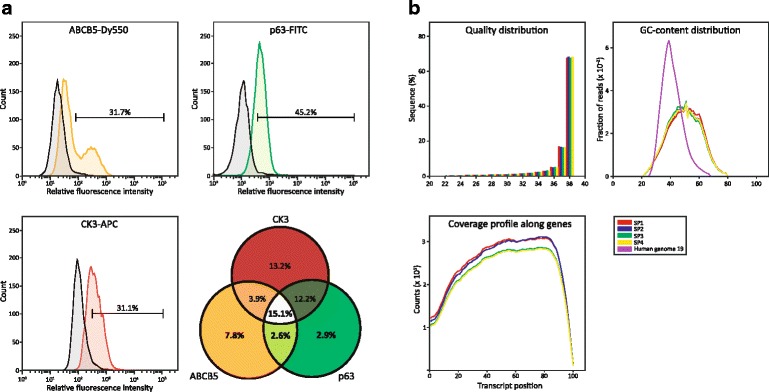
Effect of optimized procedure on the separation of subpopulations and the sequencing analysis of RNA quality. **a** Discrimination from background using top 2.5 percentile of control intensity as a cut-off is shown in representative traces for both membrane (ABCB5) and intracellular (p63 and CK3) antigens. Robust and specific sorting produced four subpopulations indicated in boldface. **b** The quality of reads was assessed by sequence quality (PHRED score), mapping coverage, and GC-content distribution

For phenotype analysis and gate setting, data on a minimum of 10,000 events were collected. For sorting, the Summit Software v4.3 (Beckman Coulter) was used, with the sort mode set as “purify”, and the gates set with reference to the isotype and FMO controls (Fig. 2a). To maximize the sample integrity, the sorting procedure was run at low pressure (20 Psi), a 100 μm sorting nozzle was used and the temperature of the sorting chamber as well as the flow line was maintained at 4 °C. A typical sorting run took 2 to 3 h to complete. Four subpopulations were sorted (SP1–4), consisting of cells with the following profiles ABCB5^+^p63^+^CK3^+^ (SP1), ABCB5^+^p63^+^CK3^−^ (SP2), ABCB5^+^p63^−^CK3^−^ (SP3), and ABCB5^−^p63^+^CK3^−^ (SP4). The subpopulations were sorted into polypropylene round bottom FACS tubes (BD Falcon) pre-filled with the sort/collect buffer. After taking an aliquot for quality control, the sorted subpopulations were pelleted, the supernatant was drained completely, and the samples were flash-frozen in liquid nitrogen. Frozen cell pellets were kept at −80 °C until RNA analysis.

### RNA Analysis

For quality control the integrity of the RNA was assayed at several steps during the staining and sorting procedure with the aid of the Aurum Total RNA kit (Bio-Rad, Copenhagen, Denmark) and the Agilent 2100 Bioanalyzer (Agilent Technologies, Glostrup, Denmark) both as prescribed by the manufacture (Fig. 1a).

After flash-freezing the transcriptomic analysis of the frozen subpopulations (SP1–4) was performed by AROS Applied Biotechnology (Arhus, Denmark) using the SMART-Seq v4 Ultra Low Input RNA Kit for cDNA and Low Input Library Prep Kit V2 for sequencing library preparation (both by Takara Bio, Otsu, Japan), and Illumina HiSeq 2000 instrument (Illumina Inc., San Diego, CA) for sequencing. The sequencing data were imported as paired-ends into CLC Genomic Workbench v9.0.1 (CLC bio, Aarhus, Denmark) to enable RNA-seq reports, including reads average PHRED score and mapping statistics against the annotated *Homo sapiens* UCSC hg19 reference genome (http://hgdownload.cse.ucsc.edu/goldenPath/hg19/bigZips/). The alignment quality was further assessed by the Qualimap v2.2 (http://qualimap.bioinfo. cipf.es/) producing the read coverage and GC content profiles [23].

## Results

### Effect of Fixation and Subsequent Cell Processing on RNA

The optimization of the procedure for RNA-seq of the ex vivo expanded and FACS sorted hLESCs was done according to the paradigm illustrated in Fig. 1a. To evaluate the effect of fixation and downstream processing on the RNA quality, the samples were analyzed at each step of the protocol (Fig. 1b). No obvious effect on the visualization of ABCB5, p63, and CK3 markers in limbal epithelial cell culture was observed when treated with either 70% ethanol or 4% formaldehyde and followed by saponin as permeabilization agent (data not shown). As indicated by the electrophoretic profiles and the quantitative assessment, the fixation with 4% formaldehyde and permeabilization with 0.1% saponin resulted in practically complete disintegration of the RNA. On the other hand, implementation of 70% ethanol as a fixative, produced practically intact RNA with RIN 9.9 ± 0.1 (max RIN = 10). During the following downstream step involving the immunostaining, the RNA integrity became only marginally affected (RIN 9.4 ± 0.3), whereas the FACS sorting had a more pronounced effect, resulting in RIN 8.4 ± 0.1. Importantly from the practical point of view, we found that freezing of the sorted cells for later RNA extraction resulted in only marginally lowered RIN (7.7 ± 0.4), thus meeting the RIN recommendation for the advanced RNA sequencing platform (RIN >7).

When comparing different subpopulations, a considerable variability in RNA yield per cell was found, ranging from 2.25 to 6.37 pg of RNA per cell, with an average of 4.59 ± 1.71 from all four subpopulations. Additionally, a positive correlation between the number of lysed cells and the integrity of produced RNA was observed (Table 1). Consequently, it was important that approximately 2 × 10^7^ cells were processed for each run so that at least 400 ng of total RNA would be harvested, which is the amount necessary to guarantee a high quality sequencing data.

**Table 1:** Quality and quantity of extracted RNA from FACS purified hLESCs subpopulations

	Profile	Cell numbers	RIN	RNA yield (pg/cell)
SP1	ABCB5^+^p63^+^CK3^+^	345 × 10^3^	8.1	6.37
SP2	ABCB5^+^p63^+^CK3^−^	94 × 10^3^	7.5	4.97
SP3	ABCB5^+^p63^−^CK3^−^	73 × 10^3^	7.3	4.75
SP4	ABCB5^−^p63^+^CK3^−^	109 × 10^3^	7.9	2.25

FACS-sorted hLESCs subpopulations from three independent experiments were pooled for RNA extaction and subsequent evaluation of RNA integrity and quantity. RIN = RNA integrity number; SP = subpopulation

### FACS Sorting of Limbal Epithelial Progenitors and Sequencing Analysis

The optimized immunostaining procedure enabled robust and specific identification of the surface and intracellular epitopes as indicated by overlays with control histograms (Fig. 2a). Based on co-expression of individual markers, four distinct subpopulations (SP1–4) could then be sorted out. As shown in the Venn diagram, the cells expressing only a single stemness-associated marker ABCB5 or p63, or combination thereof, comprised only 13.3% of the hLESC culture. The differentiation marker CK3 was expressed alone or in combination with stemness markers in 44.3% of the cells. Repertoires highlighted in bold were sorted for subsequent RNA-seq.

The performance of the sequencing was assessed by assessing the overall read quality, the mapping statistics and read coverage, and the GC content (Fig. 2b, Table 2). The average read count was 114.03 × 10^6^, which corresponds well with the expected sequencing sensitivity for genes expressed at low levels. The PHRED quality score (>80%) was higher than 30 for 80% of all base calls. For mapped reads, the average GC content was 49.25% and the GC content per sample displayed a normal distribution. After alignment, on average, 82.67% of reads were mapped in pairs to the reference genome (human genome hg19), and 86.96% of total mapped reads were mapped to exonic regions. Reads coverage profile over gene body was obtained by scaling all transcripts to 100 nt and calculating the number of reads covering each nucleotide position. The result showed uniform gene body coverage. Raw sequencing data were deposited in the Gene Expression Omnibus (GEO, http://www.ncbi.nlm.Nih.gov/geo/) under the accession number PRJNA387095 and ID 387095**.**

**Table 2:** Parameters of extracted RNA from FACS purified hLESCs subpopulations

	Read count (M)	Reads mapped in pairs (%)	GC content (%)	Reads mapped to exonic regions (%)
SP1	110.7	85.87	49.76	88.78
SP2	113.2	82.54	49.22	85.33
SP3	109.2	81.18	48.91	85.61
SP4	123	82.67	49.12	88.13

M = million; SP = subpopulation

### Statistics

The data are presented as mean ± SD from three independent experiments entailing a total of 2–4 biological replicates. One-way ANOVA (LSD) of the SPSS 24.0 package (SPSS, Chicago, IL) was used to test for differences between the samples, and *P* < 0.05 was set to discriminate statistical significance.

## Discussion

During the last decade, there has been an impressive advancement in the tools for transcriptome analysis in the scientific community and in our laboratory, from the rather simple membrane arrays allowing simultaneous analysis of a couple of hundreds of different genes [24], to more advanced microarrays enabling analysis of several hundreds of thousand genes [25]. In addition to these hybridization arrays, that are based on genes known a priori and that require a somewhat high amount of input RNA, the more advanced techniques such as deep RNA sequencing (RNA-seq), has facilitated a global analysis of the transcriptome permitting detection of alternative transcripts, with a high dynamic range, and from a smaller amount of RNA [26]. Notwithstanding, as with all techniques, the quality of the input RNA is imperative for reliable results [27].

For the sorting of heterogeneous cell populations, we rely on both extracellular and intracellular markers, and therefore need to fix and permeabilize the cells prior to staining and sorting. Formaldehyde has traditionally been used as a fixative in histology and immunochemistry due to its ability to preserve cell morphology. This is due to the formation of protein-protein and protein-nucleic acid cross-links involving methylene bridges (-CH2-) [28]. However, the use of formaldehyde as a fixative in molecular biology has been questioned, since it has been suspected to cause RNA degradation and/or making it difficult to extract RNA due to cross-linking with proteins or chemical modifications [29]. In recent years, multiple studies have indicated that non-cross-linking alcohol-based fixatives, such as ethanol, allow for the isolation of RNA with better integrity [19–21]. This is due to the fact that ethanol does not support cross-linking but allows for minor chemical modifications to the nucleic acids [8]. Our study also corroborated the superiority of ethanol, since no tangible RNA degradation was seen after the fixation and permeabilization, and during the following downstream steps, the RNA quality was preserved to support high quality sequence data.

Another major challenge in this study was to protect the RNA against the RNase-mediated degradation. The cells in our protocol were permeabilized to reveal intracellular antigens, which allowed for an increased exposure to contaminating extracellular RNase. RNase inhibitor was therefore included in all solutions following fixation and permeabilization, and rigorous cleaning was implemented to decontaminate all surfaces coming into contact with the cells. As another measure to reduce the exposure to RNase, the buffering capacity of the solutions was boosted by adding a non-phosphate HEPES system and a gentle cell detachment solution (Accumax) was implemented with the aim to minimize the cell death. All additives used in this study were previously shown to have no adverse effect on RNA integrity [11]. Since cells undergoing FACS can experience physical stress [12], RNA integrity can be further improved by decreasing the fluid pressure and/or increasing nozzle diameter [13], and keeping the cells at a lowered temperature. It is also recommended that the cells are directly sorted into RNA extraction buffer [30], however, when dealing with a large number of cells, the high volume of sheet fluid can result in undesirable dilution of RNA extraction buffer and possibly reduce RNA recovery. Thus in our study, the cells were deposited directly in the sPBS-based buffer, pelleted and frozen for storage.

Since degration of RNA will greatly affect the coverage of transcripts, it is of great importance to control quality of input material before transcriptomic profiling by assessing raw reads quality and mapping to the reference genome [31]. Before aligment, the quality of sample reads was assessed by length, GC content, quality score and duplicate sequences. After alignment, read mapping and read coverage was checked to further ensure the quality of the sequencing data [32]. Despite ongoing effort to improve the RNA sequencing quality control, it remains difficult to quantify the effect of RNA degradation on the sequencing performance. Transcript integrity number (TIN) was proposed to measure RNA degration at the transcript level [33]. Nevertheless, this measure was derived from tissue studies and further investigation is necessary to confirm its value in the cell culture scenario.

## Conclusions

Purification and characterization of progenitor cells in human corneal epithelium remains a challenge since the stem cells constitute only a small fraction (< 10%) of the cultured limbal epithelial cell population [34] and their exact immunophenotypical profile remains elusive. Thus a panel of proposed putative stem/progenitor markers are often applied to detect hLESCs. From the perspective point of view, our novel approach demonstrates the possibility of sorting corneal phenotypes based on p63, ABCB5, and CK3 for RNA-seq, which holds promise to reveal whether these markers are associated with gene activation that is characteristic of limbal stem or precursor cells. Further investigations are undoubtedly warranted to confirm utility of this approach beyond the cultured hLESCs.

## Additional file

Additional file 1: List of antibodies. (DOCX 12 kb)

## Abbreviations

ABCB5: ATP-binding cassette sub-family B member 5
CK3: Cytokeratin 3
FACS: Fluorescence-activated cell sorting
FMO: Fluorescence minus one
hLESCs: Human limbal epithelial stem cells
LSCD: Limbal stem cell deficiency
p63: Transformation-related protein 63
RIN: RNA integrity number
RNA: Ribonucleic acid
RNA-seq: RNA sequencing
SD: Standard deviation
SP: Subpopulation
TIN: Transcript integrity number
