# Supplementary material for: Maintaining RNA Integrity for Transcriptomic Profiling of Ex Vivo Cultured Limbal Epithelial Stem Cells after Fluorescence-Activated Cell Sorting (FACS)
Source: Biol Proced Online. 2017 Dec 12;19:15. doi: 10.1186/s12575-017-0065-2 (PMC5727887; doi:10.1186/s12575-017-0065-2)
Supplement: Additional file 1: — List of antibodies. (DOCX 12 kb) [file 12575_2017_65_MOESM1_ESM.docx]

| Table S1: List of antibodies | | | | |
| --- | --- | --- | --- | --- |
|  | **Catalogue number** | **Isotype** | **Clonality** | **Working dilution** |
| ABCB5-Dy550 | LS-C180816  (LifeSpan BioSciences, Seattle, WA) | Rabbit IgG | Polyclonal | 1:50 |
| P63-FITC | 167531-FITC  (US Biological, Salem, MA) | Goat IgG | Polyclonal | 1:200 |
| CK3-APC | 037615-APC  (US Biological) | Rabbit IgG | Polyclonal | 1:100 |
